# Overexpression of Telomerase Protects Human and Murine Lung Epithelial Cells from Fas- and Bleomycin-Induced Apoptosis via FLIP Upregulation

**DOI:** 10.1371/journal.pone.0126730

**Published:** 2015-05-07

**Authors:** Nissim Arish, Pazit Y. Cohen, Regina Golan-Gerstl, Zvi Fridlender, Mark Richter Dayan, Philip Zisman, Raphael Breuer, Shulamit B. Wallach-Dayan

**Affiliations:** 1 Laboratory for Lung Cellular & Molecular Biology, Institute of Pulmonary Medicine, Hadassah - Hebrew University Medical Center, Jerusalem, Israel; 2 Department of Pulmonary and Critical Care Medicine, Hospital of the University of Pennsylvania, Philadelphia, PA, United States of America; 3 Department of Emergency Medicine, Shaare Zedek Medical Center, Jerusalem, Israel; 4 Department of Pathology, Boston University School of Medicine, Boston, MA, United States of America; INSERM-Université Paris-Sud, FRANCE

## Abstract

High doses of bleomycin administered to patients with lymphomas and other tumors lead to significant lung toxicity in general, and to apoptosis of epithelial cells, in particular. Apoptosis of alveolar epithelium is an important step in the pathogenesis of bleomycin-induced pulmonary fibrosis. The Fas-FasL pathway is one of the main apoptotic pathways involved. Telomerase is a ribonucleoprotein RNA-dependent DNA polymerase complex consisting of an RNA template and a catalytic protein, telomerase reverse transcriptase (TERT). Telomerase also possess extra-telomeric roles, including modulation of transcription of anti-apoptotic genes, differentiation signals, and more. We hypothesized that telomerase overexpression affects Fas-induced epithelial cell apoptosis by an extra-telomeric role such as regulation of anti-apoptotic genes, specifically FLICE-like inhibitory protein (FLIP). Telomerase in mouse (MLE) and human (A549) lung epithelial cell lines was upregulated by transient transfection using cDNA hTERT expression vector. Telomerase activity was detected using a real-time PCR-based system. Bleomycin, and bleomycin-induced Fas-mediated apoptosis following treatment with anti-Fas activating mAb or control IgG, were assessed by Annexin V staining, FACS analysis, and confocal microscopy; caspase cleavage by Western blot; FLIP or Fas molecule detection by Western blot and flow cytometry. hTERT transfection of lung epithelial cells resulted in a 100% increase in their telomerase activity. Fas-induced lung epithelial cell apoptosis was significantly reduced in hTERT-transfected cells compared to controls in all experiments. Lung epithelial cells with increased telomerase activity had higher levels of FLIP expression but membrane Fas expression was unchanged. Upregulation of hTERT+ in human lung epithelial cells and subsequent downregulation of FLIP by shFLIP-RNA annulled hTERT-mediated resistance to apoptosis. Telomerase-mediated FLIP overexpression may be a novel mechanism to confer protection from apoptosis in bleomycin-exposed human lung epithelial cells.

## Introduction

High doses of bleomycin administered in the 1980s–1990s to patients with lymphomas and other tumors were associated with significant lung toxicity in general and apoptosis of epithelial cells in particular in 2–40% of patients, with up to 83% mortality in patients who developed lung fibrosis secondary to chemotherapy [1]. Lung toxicity has been greatly reduced in more recent reports, albeit at the cost of a reduction in cumulative dose by 75% or more, from levels >100 mg/sqm to a practical limit of approximately 25 mg/sqm today. This ceiling on cumulative dose limits the effectiveness of an important chemotherapeutic agent.

Intratracheal administration of bleomycin in mice has been widely used as an animal model mimicking side effects from treatment in lymphoma patients to study the mechanisms of lung injury, including the cycle of inflammation, and repair, and lung fibrosis [2, 3]. The pathogenesis of idiopathic pulmonary fibrosis (IPF) is typically characterized by abnormalities of alveolar structure accompanied by myofibroblast accumulation and collagen deposition in the extracellular matrix, with resulting lung scarring and inhibition of gas exchange [4]. Lung injury following bleomycin administration is manifested by epithelial cell apoptosis (programmed cell death) and evolution of fibrosis. Altered function of the Fas-FasL pathway of apoptosis in lung fibroblasts and epithelial cells has been shown to be involved in the fibrotic process [5–7]. We have shown that following bleomycin treatment of murine lung epithelial (MLE)-cells in vitro [8–10], and following in vivo treatment of C57BL/6 mice [8], both primary epithelial cells and those from a cell line become more sensitive to Fas-induced apoptosis exerted either by Fas-agonists or by activated myofibroblasts [8].

Fas (CD95/APO-1) is a 45-kDa type I transmembrane protein belonging to the tumor necrosis factor superfamily of receptors. Apoptosis is initiated when Fas receptor cross-links with FasL or agonistic anti-Fas antibodies [11–13]. However, Fas surface expression does not always correlate with Fas/FasL-induced cell death and apoptosis. Fas transduces lung myofibroblast proliferation and differentiation signals [7], and differences in sensitivity to Fas-induced apoptosis are mediated, at least in part, by FLICE-Like inhibitory protein (FLIP) expression [7] or downregulation of Fas receptor expression [14].

Telomerase is a ribonucleoprotein RNA-dependent DNA polymerase complex that consists of an RNA template and a catalytic protein, telomerase reverse transcriptase (TERT) [15]. Its main function is to maintain telomere length, resulting in attenuation of cell apoptosis and longer cell survival [16, 17]. However, emerging evidence suggests that telomerase has additional extra-telomeric roles in mediating cell survival, including anti-apoptotic functions in the presence of various cytotoxic stresses. There is evidence that telomerase, and the TERT unit in particular, might play a role in transcription [18–20], myofibroblast differentiation [21], and even protection against TRAIL-induced apoptosis [22], all independent of telomere length.

Telomere length is not the only mechanism that restricts the immortalization of many cell types. We have previously demonstrated in bleomycin-treated mouse lungs that, even when telomere length remains constant, telomerase is detected at levels that are inversely correlated with the level of lung epithelial cell apoptosis, and inhibition of telomerase with TMPYP4 increases cell death and apoptosis during evolution of lung fibrosis [23]. Moreover, treatment with a small molecule that mediates telomerase activation suppressed the development of bleomycin-induced fibrosis and accumulation of senescent cells in the lungs [24]. Ectopic expression of the human reverse transcriptase catalytic domain (hTERT) is sufficient to reconstitute telomerase enzyme activity in human [25–27] as well as mouse cells [28]. We hypothesized that hTERT overexpression in mouse and human lung epithelial cells would also involve extra-telomeric roles such as upregulation of survival gene expression, specifically FLIP, with subsequent attenuation of bleomycin- and bleomycin sensitized Fas-induced apoptosis.

## Materials & Methods

### Cell Line and Culture

The human lung alveolar epithelial cell line A549 was obtained from the American Type Culture Collection (ATCC CCL 185). Cells were cultured in Dulbecco's modified Eagle's medium (DMEM, Sigma Aldrich, St. Louis, MO, USA) supplemented with 10% heat-inactivated fetal bovine serum (Sigma Aldrich), 2 mM l-glutamine and penicillin-streptomycin (50 units/ml) (Gibco, Life Technologies/ThermoFisher Scientific, Waltham, MA, USA). The murine type II, simian vacuolating virus 40 transformed mouse lung epithelial cell line (ATCC, MLE-15) was maintained in HITES (Ham’s F12, insulin, transferin, b-estradiol and sodium selenite) medium (Biological Industries, Beit HaEmek, Israel) supplemented with 2% fetal bovine serum (Biological Industries).

### Reagents

Fluorescein isothiocyanate (FITC)-conjugated Annexin V (BD Pharmingen, San Diego, CA, USA), bleomycin (ASTA Medica, Frankfurt am Main, Germany), Dulbecco/Vogt modified Eagle’s minimal essential medium (DMEM, Sigma Aldrich), DNase (Sigma Aldrich), 4% para formaldehyde (Sigma Aldrich), and saponin buffer (Sigma Aldrich).

### Exposure of Lung Epithelial Cells to Bleomycin

MLE and A549 cells suspended in HITES (Biological Industries #06-1095-15) and F-12K (ATCC #30–2004) medium, respectively, were incubated with or without 0.1 unit-mL^-1^ of bleomycin. This bleomycin dose was selected based on previous kinetic studies in our laboratory, which have shown that at 24–72 h it induces significant, but not overwhelming, apoptosis [8–10]. After incubation, trypsin (Biological Industries) was added for adherent cell removal and the mixture was centrifuged (2536g, 10 min). The pellet was resuspended for further evaluation and Fas-induced apoptosis. Viable cells were counted using trypan blue (Sigma Aldrich).

### Apoptotic Gene Array of Bleomycin-Treated Mouse Lung Epithelial (MLE) Cells

Total cellular RNA was extracted from mouse lung epithelial cells using the Tri-Reagent Kit and instructions (Molecular Research, Inc., Cincinnati OH, USA). A mouse apoptosis pathway gene array kit (GEArray, SABiosciences/QIAGEN, Frederick, MD, USA) was used to determine the expression levels of multiple genes involved in epithelial cell apoptosis. Briefly, 1μg RNA was used as a template to generate Biotin-16-dUTP-labeled cDNA probes, according to the manufacturer’s instructions. cDNA probes were denatured and hybridized at 60°C with the SuperArray membrane, which was washed and exposed to a chemoluminescent substrate. To analyze the SuperArray membrane, we scanned the X-ray film and imported it into Adobe Photoshop (Adobe Systems, Inc., San Jose CA, USA) as a “TIFF” file. The image file was inverted. A pool of four cDNA spots for each gene was digitized using ScanAlyze software (shareware, available at http://rana.lbl.gov/EisenSoftware.htm), and normalized by subtraction of the background average intensity value of three spots containing plasmid DNA (PUC18). The average of two RPL13A spots was used as a positive control and set as the baseline value with which the signal intensity of other spots was compared. Using these normalized data, signal intensities were compared using the GEarray analyzer program (available at http://www.superarray.com).

### GeneArray Analysis

Specific cDNA fragments of 96 apoptosis-related genes were hybridized with cDNA probes synthesized from two total RNA samples corresponding to untreated (control) mouse lung epithelial cells and epithelial cells treated with 0.06mU of bleomycin (BLEO). Relative expression levels of the various genes were estimated by total RNA samples (1 μg). Lung epithelial cell RNA, with- and without bleomycin treatment, was subjected to gene SuperArray analysis. Relative expression levels of genes relevant to apoptosis were estimated by comparing signal intensities of four spots of cDNA for each relevant gene with the intensity of four spots of RPLA housekeeping gene, and then quantified by densitometry after background subtraction. The degree of gene expression after bleomycin-and saline-control treatment, as indicated by fold changes, was calculated by raw densitometry values by comparing signal intensity to RPLA 13A and then quantified by densitometry after background subtraction and determined as OD. Differentially expressed genes were identified by volcano analysis using a threshold of >2-fold changes in expression. P<0.05 was considered statistically significant.

### hTERT-Telomerase Transfection into Lung Epithelial Cell Lines

MLE and A549 cells were transfected to produce high levels of telomerase and telomerase activity as we previously detailed [29]. 0.5×10^6^ cells were incubated with a mixture of 4 mg/ml liposome (Dreamfect, OZ Biosciences, Marseille, France) and 1 μg/ml plasmids containing TERT cDNA for 1 hr (kindly provided by Prof. Varda Rotter, Weizmann Research Institute), washed, cultured with adequate medium, incubated and analyzed at 24 hr.

### Telomerase Activity

Telomerase activity was detected using the TRAPeze Telomerase detection kit (Intergen, Burlington MA, USA). The PCR-based TRAP (Telomeric Repeat Amplification Protocol) method was used, according to the kit manual and as detailed by us previously [23]. Briefly, PCR amplification was performed, and the samples were loaded and run in SDS PAGE gel. The intensity of the fluorescent signal emitted by the PCR products was determined by densitometry using a tabletop scanner (Multi-Analyst PC Version 1.1; Bio-Rad Laboratories, Hercules, CA, USA). Data were analyzed with the Fluor-S-MultiImager (Bio-Rad Laboratories). Each cDNA was amplified in triplicate, corrected to the level of GAPDH mRNA, and the median value used. Amplification was repeated with a smaller quantity of substrate if the densitometer signal was beyond the predetermined linear range.

### Fas Activation

Myofibroblast Fas activation was performed as we have previously detailed [9] using purified NA/LE DX2 mouse anti-human or Jo2 rabbit anti-mouse CD95 antibody (BD Pharmigen, Franklin Lakes, NJ, USA).

### In Vitro Detection of Apoptotic Cells by Annexin V Affinity Labeling

FITC-conjugated Annexin V (1 μg) and 5 μg/ml propidium iodide (PI) were added to 0.5×10^6^ adherent MLE and A549 cells in the culture flasks, which were then incubated (30 min), and analyzed by flow cytometry (FACStar, Becton Dickinson, Mountain View CA, USA), as described [8–10].

### Lentiviral Production and Epithelial cell Infection

Lentiviral-based vectors for RNA interference-mediated gene silencing consisted of an U6 promoter for expression of short hairpin RNAs (shRNAs) under the control of SV40 promoter for monitoring transduction efficiency. The oligonucleotide used to produce V3LHS_346941 Dharmacon plasmid-based CFLAR (c-FLIP) shRNA was 5'- TTGTCTTCAGGTCTATTCT-3' cloned into the SV vector using AgeI and BamHI restriction sites. Lentiviral particles were produced in 293T (human embryonic kidney cells), co-transfected by the calcium phosphate method with the above plasmid plus plasmid coding for the envelope and packaging systems (VSV-G and D8.9, respectively). Endogenous protein knock-down of FLIP protein expression was assessed by Western blot.

### Fas and FLIP Expression Assayed by Flow Cytometry

As we detailed previously [7], cell surface expression of Fas, and intracellular FLIP was assessed by indirect immuno-fluorescence and analyzed by flow cytometry. Briefly, 0.5×10^6^ cells were washed with FACS medium (3% FCS in PBSX1) for Fas staining, and with permeabelazing saponin medium for FLIP staining, and incubated on ice for 45 min with anti-Fas or anti FLIP conjugated mAb (1μg/100 μl FACS or saponin buffer). After 30 min, the cells were washed and analyzed by flow cytometry using a FACStar.

### Western Blot

As we have detailed [29]. Lung epithelial cell protein was blotted (in similar amounts), incubated with anti-FLIP (Stressgen Biotechnology, Victoria BC, Canada) or anti-caspase 8/caspase-3 antibodies (1:1000) (Cell Signaling Technology, Danvers MA, USA), re-incubated for 1 h in the appropriate horseradish peroxidase-conjugated (HRP) antiserum (1:2,500 dilution, Jackson Laboratories, Bar Harbor, ME, USA), and analyzed with the ECL detection system (Bio-Rad, Haifa, Israel).

### Statistical Analysis

Analysis of variance was performed with the Kruskal-Wallis test for nonparametric data. When Kruskal-Wallis tests of comparability were statistically significant, Mann-Whitney comparisons with Holms sequential Bonferroni-corrected p values were performed. In order to determine whether relative telomerase activity was consistently greater or less than baseline, the data were dichotomized. One-sample Chi-squared test was performed, comparing the observed distribution with an expected random distribution. p< 0.05 was considered significant.

### Theory

Telomerase-TERT influences FLIP expression, which in turn modulates cell apoptosis. We hypothesized that by upregulating epithelial cell telomerase and FLIP expression, it may be possible to protect epithelial cells against bleomycin- and Fas-induced apoptosis, and thus attenuate lung injury and fibrosis in general, and in cancer patients treated with bleomycin, in particular.

## Results

### Apoptotic Gene Expression in Mouse Lung Epithelial (MLE) Cells after Bleomycin Treatment

**Fig 1:**
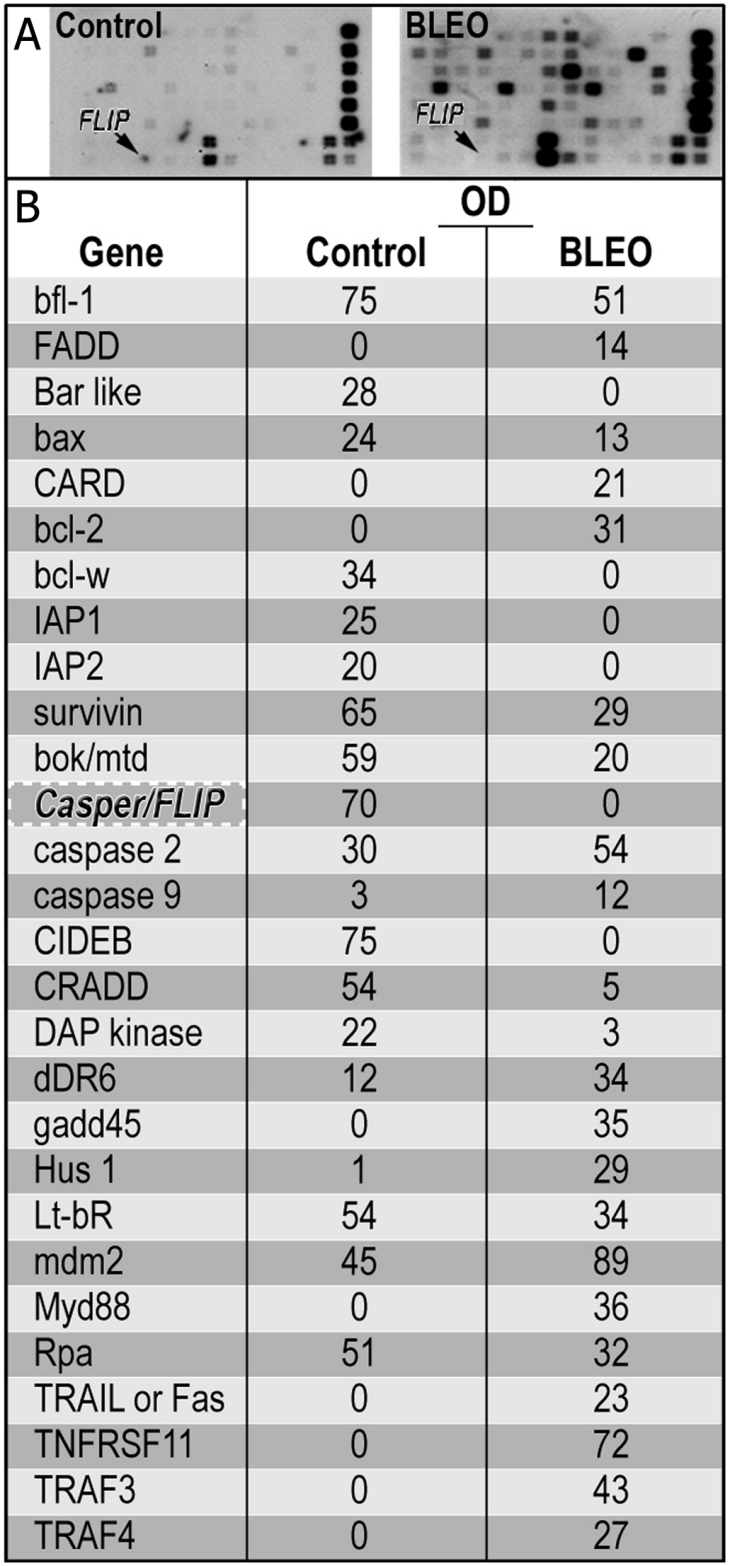
Apoptotic gene expression in MLE cells after bleomycin treatment. (A) Specific cDNA fragments of 96 apoptosis-related genes were hybridized with cDNA probes synthesized from two total RNA samples corresponding to untreated (Control) mouse lung epithelial cells and epithelial cells treated with 0.06mU of bleomycin for 72h (BLEO). (B) Relative expression levels of genes relevant to apoptosis. Among other survival genes, FLIP was shown to be downregulated after exposure to bleomycin. The degree of gene expression after bleomycin-and saline-control treatment, as indicated by fold changes, was calculated by raw densitometry values by comparing signal intensity to RPLA 13A and then quantified by densitometry after background subtraction and determined as OD.

We have previously shown that bleomycin sensitizes MLE cells to Fas-induced apoptosis [8–10], possibly enabling their cell death by FasL^+^ myofibroblasts [8]. In this study we further analyzed the molecular mechanisms of these phenomena by assessing apoptotic primary lung epithelial cell gene expression after bleomycin treatment of mouse lung epithelial (MLE) cells, using pathway-specific array analysis. Four housekeeping genes and 96 genes among those involved in epithelial cell apoptosis were compared in cells that were treated, or not, with bleomycin for 72 h as described in Materials and Methods. We found (Fig 1, table and inserts) that bleomycin upregulates genes that are known to appear after cell exposure to oxidative stress and DNA damage such as GADD45, mdm-2, and bcl-2; genes involved in the intrinsic apoptotic pathway such as caspase-9; genes involved in the extrinsic pathway from the TNF receptor (TNFr) family; and associated molecules such as TRAIL, TRAFs, TNFsf, and FADD. However, many genes that regulate apoptosis and confer survival, such as ATM, which is known to regulate telomere length, as well as FLIP/casper, IAPs, survivin, and bcl-w, were downregulated. We have chosen to focus on the survival gene that encodes the FLIP molecule and assess effects of its ectopic upregulation in mouse and human epithelial cell lines.

### Decreased Bleomycin-Induced Apoptosis in hTERT-Transfected Mouse-Lung Epithelial (MLE) Cells

**Fig 2:**
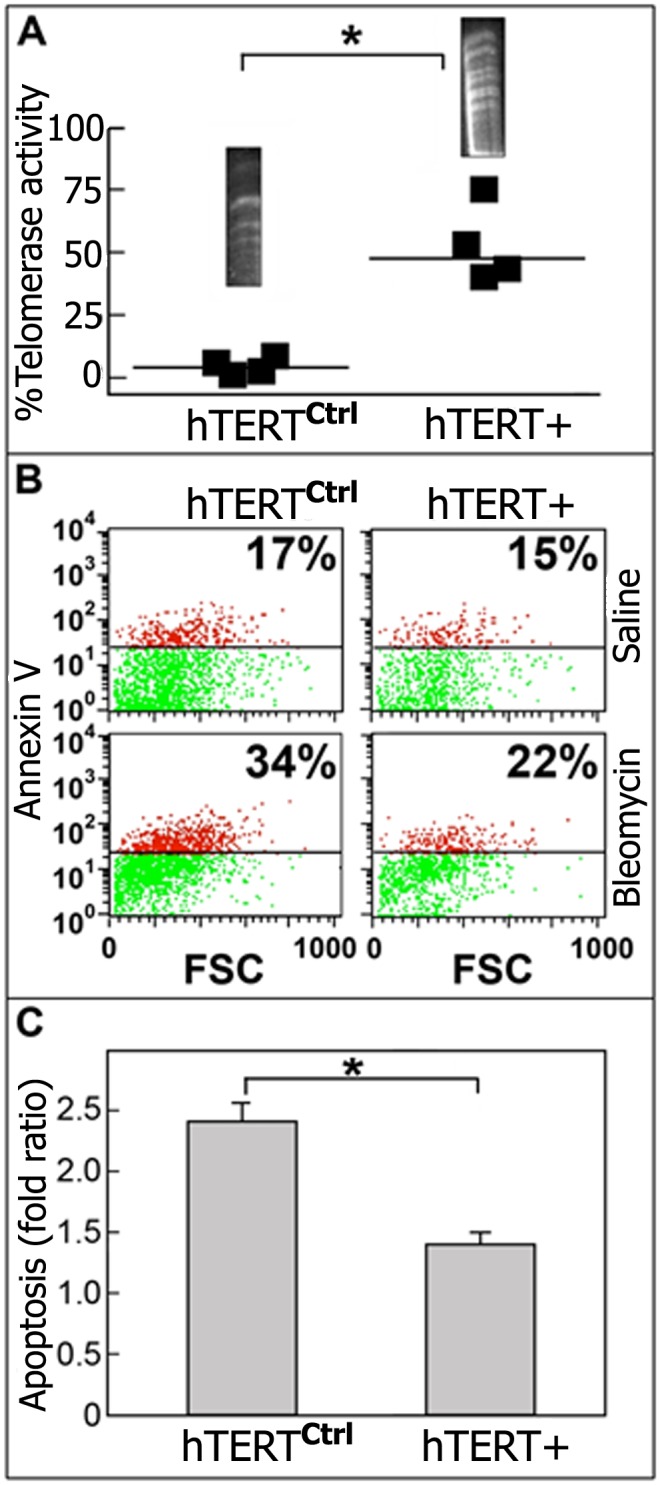
Decreased bleomycin-induced apoptosis in hTERT transfected MLE cells. (A) PCR-based telomerase activity in Mouse-Lung Epithelial (MLE) cells transfected with hTERT^+^ or control hTERT (hTERT^ctrl^) expression vectors. hTERT^+^ and hTERT^ctrl^ MLE cells were exposed to bleomycin (0.06mU) or control saline. (B) Flow cytometry analysis demonstrating decreased Annexin V staining in bleomycin-treated hTERT^+^ compared to hTERT^ctrl^ cells. (C) Bar diagram representing the fold ratio (bleomycin/saline) of MLE cell apoptosis in hTERT^+^ vs. hTERT^ctrl^ MLE cells (n = 4, *p<0.05).

Forced in vitro telomerase downregulation in MLE cells was previously shown by us to be associated with higher levels of bleomycin-induced apoptosis [23]. In this study we aimed to assess whether telomerase can directly protect bleomycin-treated lung epithelial cells from apoptosis. To this end, MLE cells were transfected with hTERT cDNA expression vector and control cDNA, and were further exposed to bleomycin or control saline for 24 h. Cells were collected and resuspended. Viable cells were counted using trypan blue in order to exclude necrotic cells. A similar number of viable bleomycin-treated and untreated cells were taken for evaluation of telomerase activity as we previously detailed [23]. Telomerase activity, assessed by the PCR-based TRAP method, was increased in hTERT cDNA-transfected cells (hTERT^+^) compared with control (hTERT^ctrl^) cells in four independent experiments (Fig 2A) with significant results (*p<0.02). By specific upregulation of telomerase in this study we directly show the role of telomerase in protecting MLE cells from bleomycin-induced apoptosis (Fig 2). As assessed by flow cytometry analysis of Annexin V binding, we initially show that the spontaneous apoptosis of MLE cells is not changed following upregulation of hTERT (Fig 2B), hTERT^ctrl^ (17%) vs. hTERT^+^ (15%) in saline treatment. However, MLE cells transfected with hTERT demonstrated a 1.5 fold decrease in bleomycin-induced apoptosis, from 34% to 22%. This experiment was independently repeated three times and the results, which are presented graphically (Fig 2C), were statistically significant (*p<0.05).

### Decreased Fas-Induced Apoptosis in hTERT^+^-Transfected MLE Cells

**Fig 3:**
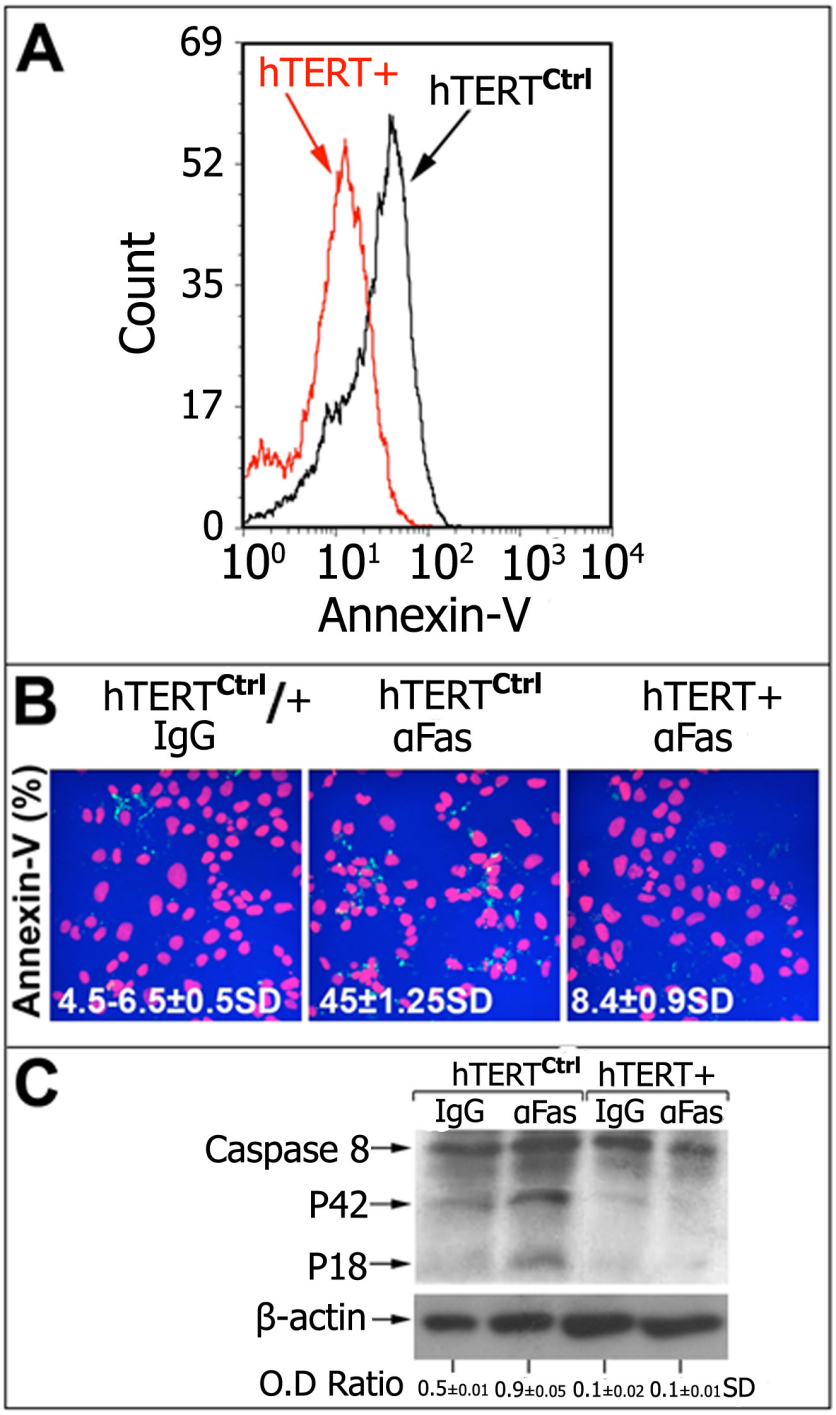
Decreased Fas-induced apoptosis in hTERT^+^ vs. hTERT^ctrl^ transfected MLE cells. MLE cells were transfected with hTERT or control expression vector, and exposed to Fas or control IgG mAb (10 μg/ml, 24h). (A) Histogram plot and (B) confocal microscopy of Annexin V staining (blue) and PI (red) in hTERT^+^ vs. hTERT^ctrl^ cells showing decreased Annexin V staining in hTERT^+^ when compared to hTERT^ctrl^ transfected cells (MLE). Inserted numbers represent the percentage of Annexin V-stained cells (blue) among total cells in the field (red) with standard deviation (SD). 10–15 fields were counted. (C) Western blots of caspase-8 cleavage into p42 and p18 subunits. Cleaved/uncleaved-caspase-8 optical density (OD) ratios are presented, showing decreased caspase 8 cleavage in hTERT^+^ when compared to the hTERT^ctrl^-transfected MLE cell line. Representative results of two different experiments with similar results.

We further determined whether the upregulation of telomerase (hTERT^+^) can protect bleomycin-treated MLE cells from sensitization to Fas-induced apoptosis (Fig 3). Apoptosis of hTERT^+^ vs. hTERT^ctrl^ in MLE cells was detected in three different assays, each repeated twice, using flow cytometry of Annexin V binding by FACS (Fig 3A) or by confocal microscopy (Fig 3B) following exposure of bleomycin-treated MLE cells to Jo2 anti-Fas mAb vs. control IgG (10μg/48h). Apoptosis was detected in only 4.5% of hTERT^+^ MLE cells; however, 45% of hTERT^ctrl^ MLE cells were apoptotic. Apoptosis was then further assessed using Western blot of caspase-8 cleavage, which showed that hTERT^+^ MLE cells significantly decreased the cleavage of caspase 8 to the p42 and p18 subunits in comparison with hTERT^ctrl^ MLE cells. The standard deviation (SD) is presented (Fig 3C).

### Levels of Fas-Death Receptor are Similar in hTERT^+^ and Control-Transfected MLE Cells

**Fig 4:**
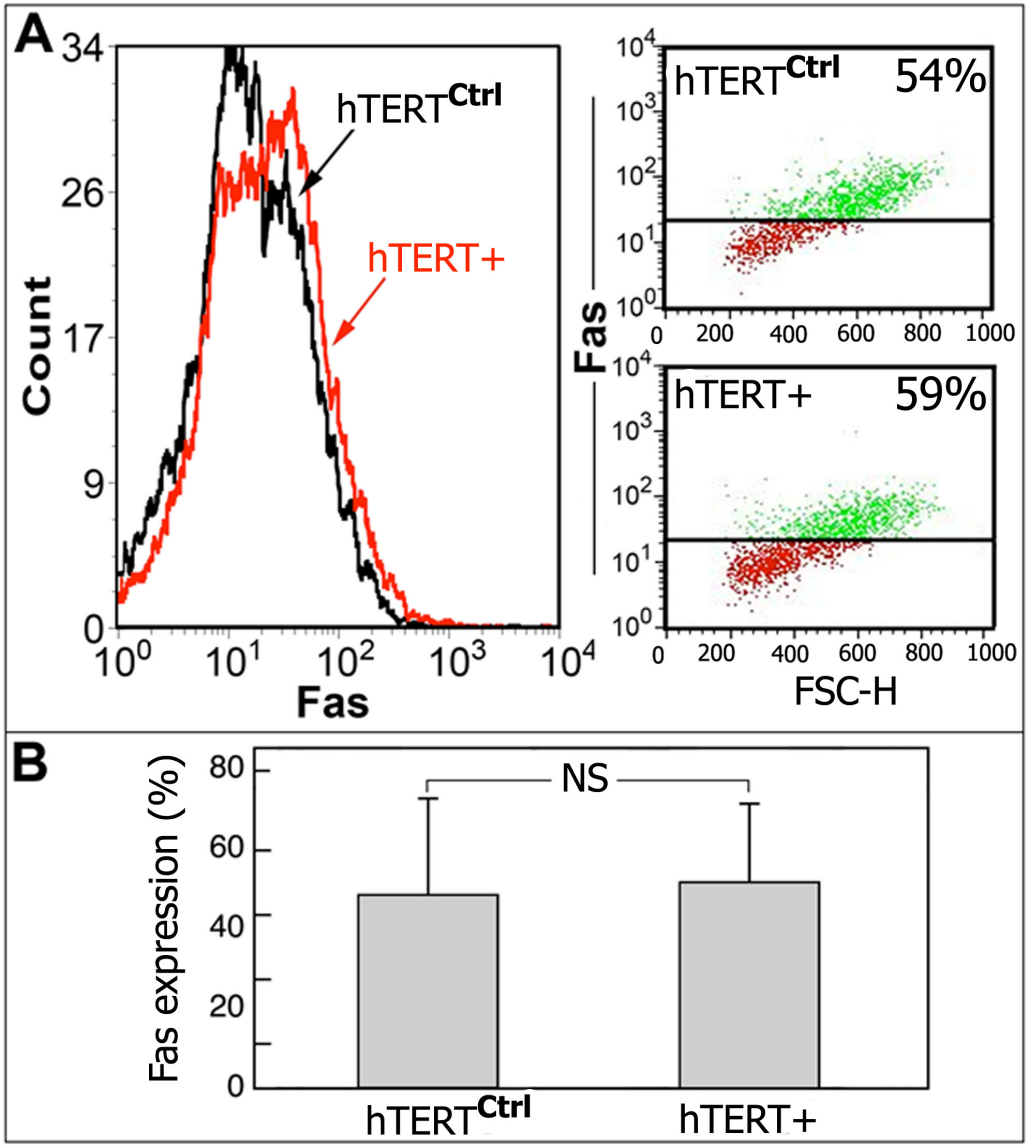
Fas expression is unchanged in hTERT^+^-transfected MLE cells. (A) Flow cytometry analysis (histogram and dot plots) showing similar Fas expression in hTERT^+^ vs. hTERT^ctrl^ MLE cells. (B) Graphic presentation of FACS analysis from three independent experiments (n = 4).

We have shown previously that protection from telomere loss is not the mechanism by which telomerase protects MLE cells from apoptosis [23]; therefore, we initially assessed possible changes in Fas-death expression as an alternative mechanism known to disturb apoptosis [30]. We found that neither the level of Fas expression, shown by FACS histogram-plot, nor the percentage of cells expressing Fas, shown by dot-plot, changed following transfection and overexpression of telomerase (Fig 4A, hTERT^+^ vs. hTERT^ctrl^, respectively). These results were confirmed in four other independent experiments, represented graphically; showing that there was no significant difference between groups (n = 4) (Fig 4B).

### FLIP is Upregulated in hTERT^+^-Transfected Mouse Lung MLE Cells

**Fig 5:**
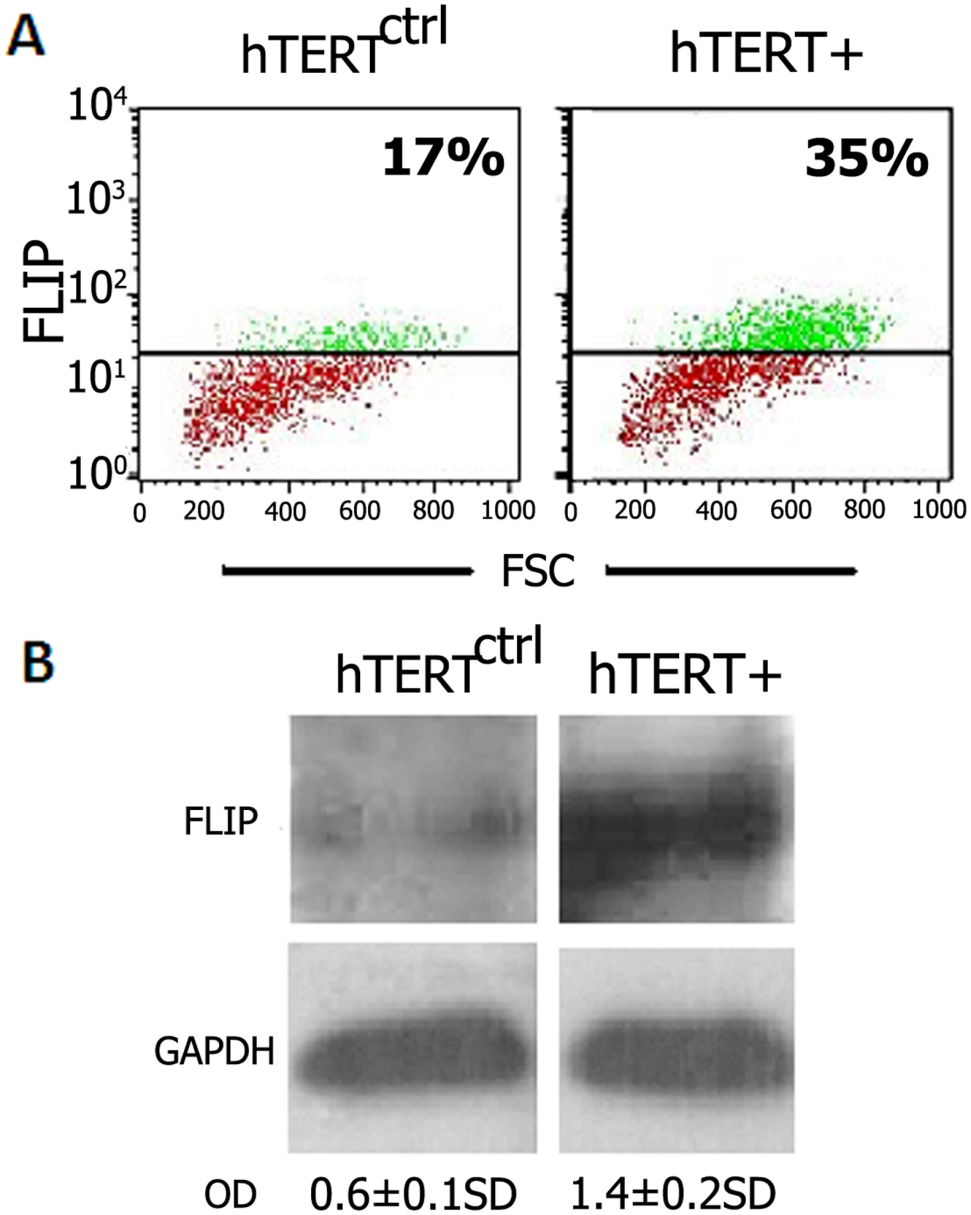
FLIP is upregulated in hTERT^+^ vs. hTERT^ctrl^ transfected MLE cells. (A) Flow cytometry dot plots of FLIP expression, and (B) Western blot using anti-FLIP mAb in hTERT^+^ vs. hTERT^ctrl^ (control) transfected cells. OD ratios showing increased FLIP in hTERT^+^ mouse lung MLE epithelial transfected cells are presented. Representative results of two different experiments for each assay, with similar results.

We showed previously that FLIP is upregulated in MLE cells and fibroblasts, and that FLIP upregulation diverts Fas-induced lung myofibroblast apoptosis towards proliferation [7]. In this study we assessed whether telomerase-TERT overexpression is associated with upregulation of FLIP levels. FACS flow cytometry analysis revealed that following hTERT^+^ vs. control-cDNA transfection, FLIP molecule expression on MLE cells increased from 17% to 35% (Fig 5A, hTERT^+^ vs. hTERT^ctrl^, respectively). These results were further confirmed by Western blot (Fig 5B).

### hTERT^+^-Transfected Human Lung Epithelial (A549) Cells Upregulate FLIP Levels and Acquire Resistance to Fas-induced Apoptosis

**Fig 6:**
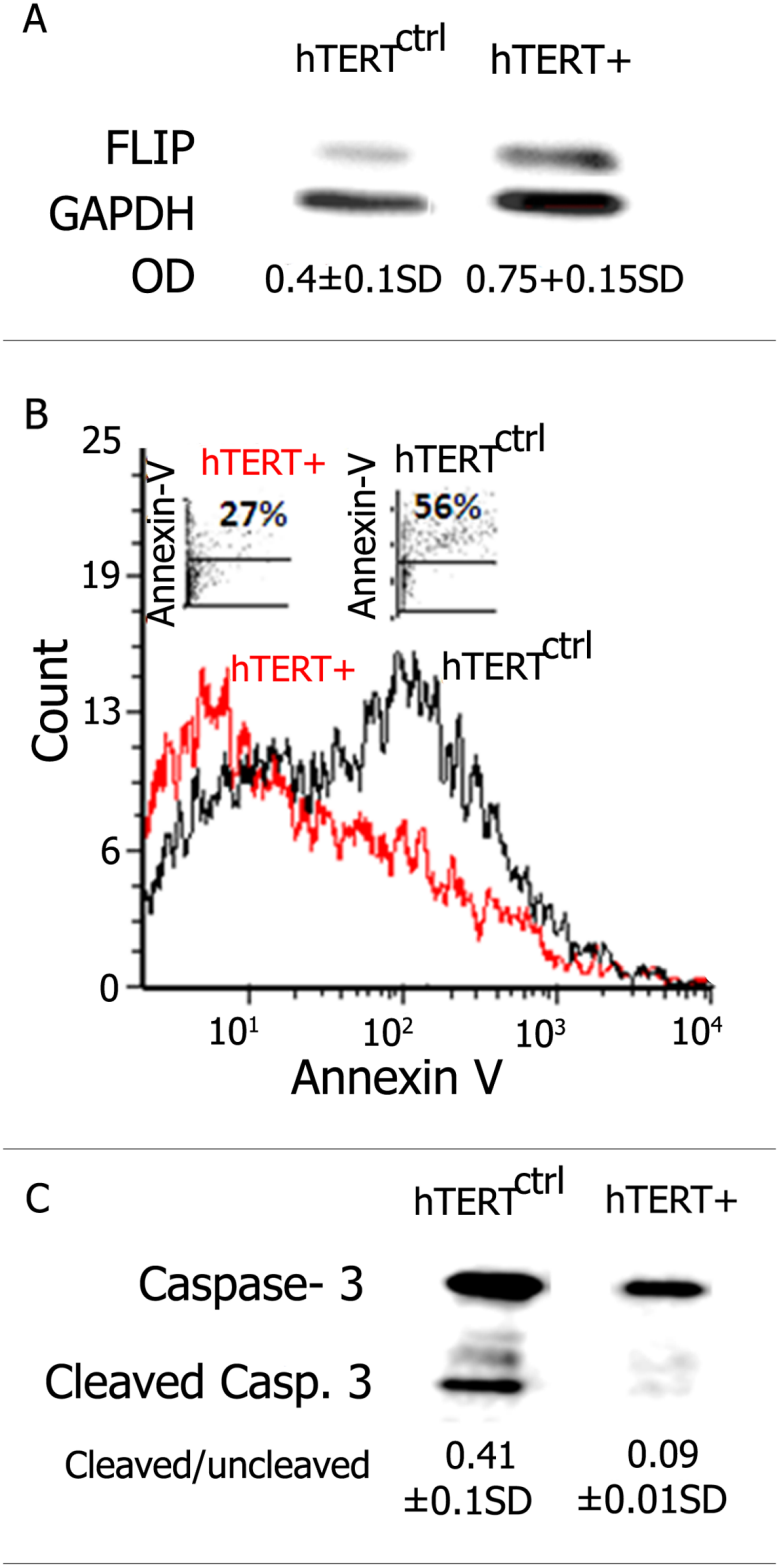
hTERT^+^-transfected human lung epithelial A549 cells upregulate FLIP levels and acquire resistance to Fas induced apoptosis. (A) Western blot of FLIP expression using anti-FLIP mAb in hTERT^ctrl^ (control) vs. hTERT^+^ transfected cells from a human lung epithelial cell line (A549). Optical densities showing increased FLIP in hTERT^+^ human lung A549 epithelial transfected cells are presented (OD). (B) Histogram plots of flow cytometry analysis showing decreased Annexin V staining in hTERT^+^ (red) when compared to hTERT^ctrl^ (black) transfected cells (A549). (C) Western blot showing decreased caspase 3 cleavage in hTERT^+^, when compared to the hTERT^ctrl^ transfected A549 cell line. Cleaved/uncleaved caspase 3 OD ratios are presented. Representative results of two different experiments for each assay, with similar results.

We then confirmed results of the TERT effect on Fas and FLIP expression, and on human lung epithelial cell susceptibility to Fas-induced apoptosis. hTERT was introduced into an A549 lung epithelial cell line in three independent transient transfections. Western blot analysis revealed that, following hTERT^+^ vs. control-cDNA transfection, FLIP molecule expression was doubled from an OD of 0.4 in hTERT^ctrl^ to 0.75 in hTERT^+^ (Fig 6A). Fas levels on A549 cells following hTERT^+^ transfection were unchanged (not shown). hTERT-transfected A549 cells were then subjected to bleomycin and treated with agonist human anti-Fas DX2 mAb (10μM, 48h). Apoptosis of hTERT^ctrl^ vs. hTERT^+^ in A549 cells, following exposure to DX2 anti-Fas mAb was detected by two different assays performed twice. Flow cytometry analysis of Annexin V binding demonstrates that only half (27%) the fraction of hTERT^+^; compared to 55% of hTERT^ctrl^ A549 cells were apoptosized (Fig 6B). These results were further confirmed by the decrease in the cleaved/uncleaved caspase-3 OD-ratio from 0.45, to only 0.09, as detected by Western blot, in hTERT^+^, compared to hTERT^ctrl^ A549 cells, respectively (Fig 6C).

### Downregulation of FLIP Levels Annuls hTERT-Mediated Resistance to Fas-Induced Apoptosis in Human Lung Epithelial A549 Cells

**Fig 7:**
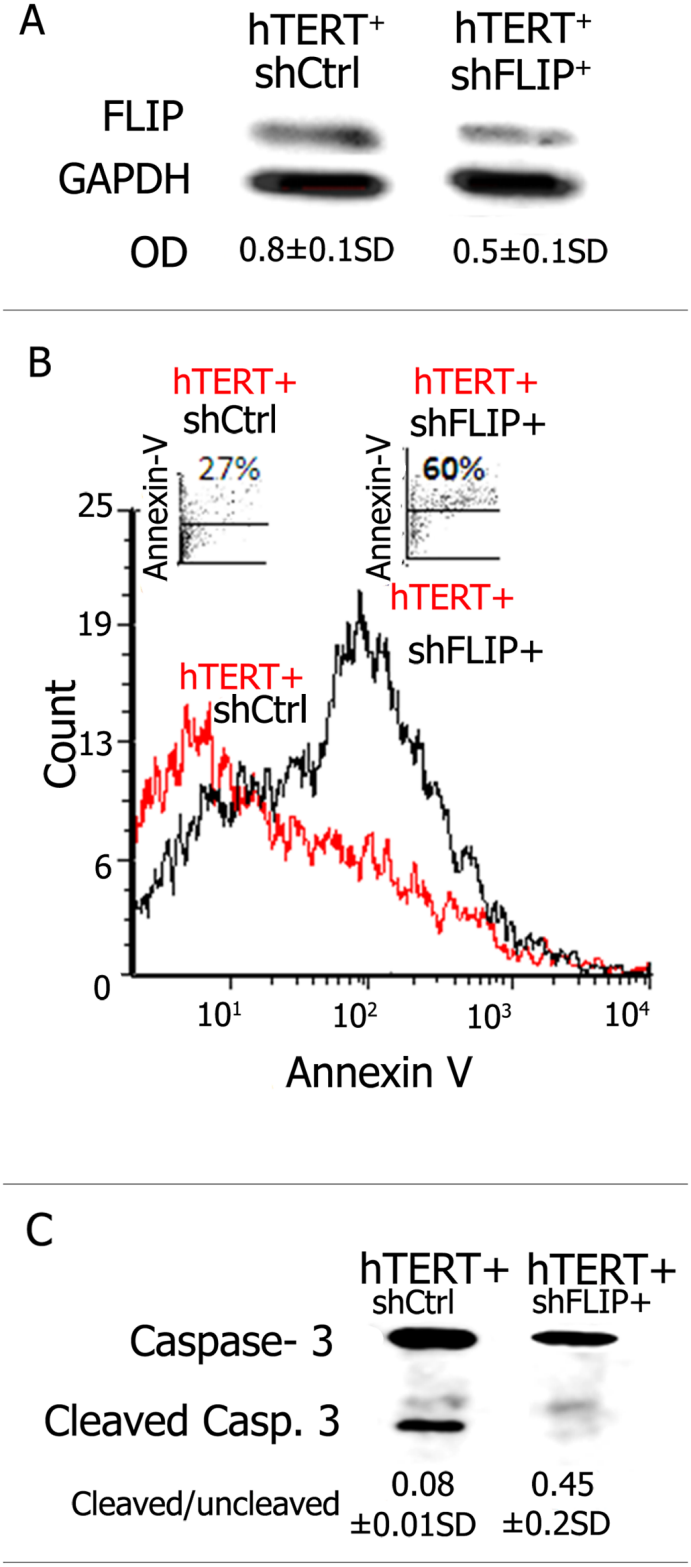
Downregulation of FLIP levels annuls hTERT-mediated resistance to Fas-induced apoptosis in human lung epithelial A549 cells. (A) Western blot of FLIP expression using anti-FLIP mAb in an hTERT+shRNA^Ctrl^ (sh-scrambled/control) vs. an hTERT+shFLIP-RNA transfected-A549 cell line. OD ratios show decreased FLIP expression in hTERT^+^ A549 cells following further transfection with shFLIP when compared to those that were further transfected with control shRNA. (B) Histogram plot of Annexin V staining and (B) Western blot of caspase 3 cleavage in hTERT^+^ human lung epithelial A549 cells after infection with (hTERTshCtrl) and hFLIP-RNA lentiviral vector (hTERTshFLIP) and subjection to agonist Fas DX2 mAb. Representative results of two different experiments for each assay, with similar results showing increased apoptosis in hTERT^+^ A549 cells transfected with shFLIP-RNA.

We then assessed, by modulation of FLIP expression in hTERT-transfected A549 cells, whether hTERT-mediated enhanced FLIP expression is significant for the protection against apoptosis. To this end we further infected hTERT^+^ and hTERT^ctrl^ A549 cells with specific lentiviruses carrying shRNA-FLIP, or the scrambled control sequence shRNA-Ctrl, and assessed induction of apoptosis following cell exposure to bleomycin and treatment with agonist anti-Fas DX2 mAb (10μM, 48h). Using Western blot analysis we found that FLIP expression decreases in shFLIP-infected hTERT^+^ A549 cells (Fig 7A). Flow cytometry analysis of Annexin V staining (Fig 7B) and Western blot of caspase-3 cleavage (Fig 7C) revealed that, when compared to control A549 cells (hTERT^+^shCtrl^+^), downregulation of FLIP in hTERT-transfected A549 cells (hTERT^+^shFLIP^+^) restored susceptibility to Fas-induced apoptosis. These results suggest that FLIP is related to the anti-apoptotic activity of hTERT.

## Discussion

Apoptosis of alveolar lung epithelial cells, particularly apoptosis that is mediated by bleomycin and by bleomycin-sensitized Fas-death pathway, has been implicated as an initial event leading to pulmonary fibrosis in mice following bleomycin intratracheal instillation [5, 8, 31]. Bleomycin causes apoptosis of lung epithelial cells in vitro, accompanied by accumulation of intracellular reactive oxygen species [8–10] in a dose-response manner via a decrease in telomerase activity [23], a decrease in intracellular glutathione content [8–10], and an increased expression of cell surface CD95/Fas with enhanced sensitization to Fas-induced apoptosis [8].

We have previously demonstrated that telomerase might play a role in the protection of epithelial cells from bleomycin-induced apoptosis during the acute stages of lung fibrosis in mice. We showed that inhibition of telomerase with TMPYP4, increased epithelial cell death and apoptosis [23]. Following bleomycin exposure, MLE cells downregulate TERT [23] as well as FLIP (Fig 1) levels. In a previous study we have shown that upregulation of FLIP may play an important role in fibroblast resistance to Fas-induced apoptosis [7]. We further show in the current study that mouse and human lung epithelial cells with forced overexpression of hTERT had greater protection from bleomycin- and bleomycin sensitized Fas-induced apoptosis. Moreover, when hTERT is ectopically overexpressed, FLIP expression levels are increased in mouse and human lung epithelial cells. Of note, although this study aims to address the role of TERT in extratelomeric roles of telomerase, human TERT expression was previously extensively assessed in mouse cells and was found to be effective in activation of mouse telomerase and telomere elongation [28].

Dudognon at el also demonstrated that TERT can attenuate TRAIL (cell-death receptor)-induced apoptosis, independent of telomere length [22]. This function of telomerase can be mediated by its action in the regulation of anti-apoptotic and growth-controlling genes, independent of telomere functions [19, 32, 33]. FLIP overexpression has been proposed to lead to lung cell resistance to apoptosis [7], and a decrease in its activity has been related to lung cell death [7]. FLIP has also been detected by us and others in fibrotic lung primary epithelial cells at specific stages of extensive lung fibrosis [7, 34, 35]. In parallel, we have found that, at these stages, telomerase activity is increased in lung epithelial cells of bleomycin-treated mouse lungs [23]. Therefore, there is a possibility that FLIP could contribute to telomerase-mediated epithelial cell survival. FLIP expression may thus be a means of TERT protection from cell death and apoptosis. In support of this hypothesis, hTERT deficiency may contribute to epithelial cell apoptosis and subsequent lung fibrosis, as previously reported, suggesting that mutations in essential genes coding for the enzyme telomerase are the most commonly identified genetic risk factors in IPF [36]. Degryse et al have performed a thorough study showing that fibrosis develops in a similar manner in both TERT-deficient and wild-type mice [37]. Nevertheless, it will be of interest to specifically assess TERT deficiency in lung epithelial cells in vivo and, in particular, the benefits of its overexpression as opposed to its deficiency in the injured lung. This relationship was previously shown in the injured liver of TERT-deficient mice [38]. However, since telomere maintenance by telomerase is known to affect alveolar epithelial cell integrity [39], we suggest that telomere maintenance may be one of the protective effects of telomerase upregulation following bleomycin treatment.

The mechanism by which TERT upregulates FLIP levels is still unknown, and warrants further study. It may be possible, as in the case with FasL expression in bone marrow mesenchymal stem cells [32], that TERT expression in lung epithelial cells contributes to FLIP transcription.

Specific overexpression of telomerase and FLIP in lung primary epithelial cells may mediate attenuation of their apoptosis and subsequent lung fibrosis. This understanding may pave the way for design of a new approach for therapeutic intervention with bleomycin in patients with lymphomas, and control of the debilitating side effects now seen.

## References

[1] Bloor AJ, Seale JR, Marcus RE. Two cases of fatal bleomycin pneumonitis complicating the treatment of non-Hodgkin's lymphoma. Clin Lab Haematol. 1998;20(2): 119–121. Epub 1998/07/29. 9681223 10.1046/j.1365-2257.1998.00100.x

[2] Adamson IY, Bowden DH. The pathogenesis of bloemycin-induced pulmonary fibrosis in mice. Am J Pathol. 1974;77(2): 185–197. 4141224 PMC1910906

[3] Izbicki G, Segel MJ, Christensen TG, Conner MW, Breuer R. Time course of bleomycin-induced lung fibrosis. Int J Exp Pathol. 2002;83(3): 111–119. 12383190 10.1046/j.1365-2613.2002.00220.x PMC2517673

[4] Phan SH. The myofibroblast in pulmonary fibrosis. Chest. 2002;122(6 Suppl): 286S–289S. 12475801 10.1378/chest.122.6_suppl.286s

[5] Kuwano K, Hagimoto N, Kawasaki M, Yatomi T, Nakamura N, Nagata S, et al Essential roles of the Fas-Fas ligand pathway in the development of pulmonary fibrosis. J Clin Invest. 1999;104(1): 13–19. 10393694 10.1172/JCI5628 PMC408400

[6] Kuwano K, Kaneko Y, Hagimoto N, Kawasaki M, Kunitake R, Tanaka T, et al Expression of B7-1, B7-2, and interleukin-12 in anti-Fas antibody-induced pulmonary fibrosis in mice. Int Arch Allergy Immunol. 1999;119(2): 112–119. 10394102 10.1159/000024185

[7] Golan-Gerstl R, Wallach-Dayan SB, Zisman P, Cardoso WV, Goldstein RH, Breuer R. Cellular FLICE-like inhibitory protein deviates myofibroblast fas-induced apoptosis toward proliferation during lung fibrosis. Am J Respir Cell Mol Biol. 2012;47(3): 271–279. 10.1165/rcmb.2010-0284RC 22582174 PMC5460908

[8] Golan-Gerstl R, Wallach-Dayan SB, Amir G, Breuer R. Epithelial cell apoptosis by fas ligand-positive myofibroblasts in lung fibrosis. Am J Respir Cell Mol Biol. 2007;36(3): 270–275. 16990614 10.1165/rcmb.2006-0133OC

[9] Wallach-Dayan SB, Golan-Gerstl R, Breuer R. Evasion of myofibroblasts from immune surveillance: a mechanism for tissue fibrosis. Proc Natl Acad Sci U S A. 2007;104(51): 20460–20465. 18077384 10.1073/pnas.0705582104 PMC2154453

[10] Wallach-Dayan SB, Izbicki G, Cohen PY, Gerstl-Golan R, Fine A, Breuer R. Bleomycin initiates apoptosis of lung epithelial cells by ROS but not by Fas/FasL pathway. Am J Physiol Lung Cell Mol Physiol. 2006;290(4): L790–L796. 16306138 10.1152/ajplung.00300.2004

[11] Itoh N, Yonehara S, Ishii A, Yonehara M, Mizushima S, Sameshima M, et al The polypeptide encoded by the cDNA for human cell surface antigen Fas can mediate apoptosis. Cell. 1991;66(2): 233–243. 1713127 10.1016/0092-8674(91)90614-5

[12] Jelaska A, Korn JH. Anti-Fas induces apoptosis and proliferation in human dermal fibroblasts: differences between foreskin and adult fibroblasts. J Cell Physiol. 1998;175(1): 19–29. 9491777 10.1002/(SICI)1097-4652(199804)175:1<19::AID-JCP3>3.0.CO;2-F

[13] Oehm A, Behrmann I, Falk W, Pawlita M, Maier G, Klas C, et al Purification and molecular cloning of the APO-1 cell surface antigen, a member of the tumor necrosis factor/nerve growth factor receptor superfamily. Sequence identity with the Fas antigen. J Biol Chem. 1992;267(15): 10709–10715. 1375228

[14] Buhling F, Wille A, Rocken C, Wiesner O, Baier A, Meinecke I, et al Altered expression of membrane-bound and soluble CD95/Fas contributes to the resistance of fibrotic lung fibroblasts to FasL induced apoptosis. Respir Res. 2005;6: 37 15833141 10.1186/1465-9921-6-37 PMC1087885

[15] Cong YS, Wright WE, Shay JW. Human telomerase and its regulation. Microbiology and molecular biology reviews: MMBR. 2002;66(3): 407–425, table of contents. 12208997 10.1128/MMBR.66.3.407-425.2002 PMC120798

[16] Hamad NM, Banik SS, Counter CM. Mutational analysis defines a minimum level of telomerase activity required for tumourigenic growth of human cells. Oncogene. 2002;21(46): 7121–7125. 12370834 10.1038/sj.onc.1205860

[17] Ren JG, Xia HL, Tian YM, Just T, Cai GP, Dai YR. Expression of telomerase inhibits hydroxyl radical-induced apoptosis in normal telomerase negative human lung fibroblasts. FEBS Lett. 2001;488(3): 133–138. 11163759 10.1016/s0014-5793(00)02397-8

[18] Mondello C, Scovassi AI. Telomeres, telomerase, and apoptosis. Biochemistry and cell biology = Biochimie et biologie cellulaire. 2004;82(4): 498–507. 15284903 10.1139/o04-048

[19] Smith LL, Coller HA, Roberts JM. Telomerase modulates expression of growth-controlling genes and enhances cell proliferation. Nat Cell Biol. 2003;5(5): 474–479. 12717449 10.1038/ncb985

[20] Martinez P, Blasco MA. Telomeric and extra-telomeric roles for telomerase and the telomere-binding proteins. Nat Rev Cancer. 2011;11(3): 161–176. 10.1038/nrc3025 21346783

[21] Liu T, Hu B, Chung MJ, Ullenbruch M, Jin H, Phan SH. Telomerase regulation of myofibroblast differentiation. Am J Respir Cell Mol Biol. 2006;34(5): 625–633. 16424384 10.1165/rcmb.2005-0252OC PMC2644224

[22] Dudognon C, Pendino F, Hillion J, Saumet A, Lanotte M, Segal-Bendirdjian E. Death receptor signaling regulatory function for telomerase: hTERT abolishes TRAIL-induced apoptosis, independently of telomere maintenance. Oncogene. 2004;23(45): 7469–7474. 15326479 10.1038/sj.onc.1208029

[23] Fridlender ZG, Cohen PY, Golan O, Arish N, Wallach-Dayan S, Breuer R. Telomerase activity in bleomycin-induced epithelial cell apoptosis and lung fibrosis. Eur Respir J. 2007;30(2): 205–213. 17504800 10.1183/09031936.00009407

[24] Le Saux CJ, Davy P, Brampton C, Ahuja SS, Fauce S, Shivshankar P, et al A novel telomerase activator suppresses lung damage in a murine model of idiopathic pulmonary fibrosis. PLoS One. 2013;8(3): e58423 10.1371/journal.pone.0058423 23516479 PMC3597721

[25] Vaziri H, Benchimol S. Reconstitution of telomerase activity in normal human cells leads to elongation of telomeres and extended replicative life span. Curr Biol. 1998;8(5): 279–282. 9501072 10.1016/s0960-9822(98)70109-5

[26] Counter CM, Meyerson M, Eaton EN, Ellisen LW, Caddle SD, Haber DA, et al Telomerase activity is restored in human cells by ectopic expression of hTERT (hEST2), the catalytic subunit of telomerase. Oncogene. 1998;16(9): 1217–1222. 9528864 10.1038/sj.onc.1201882

[27] Bodnar AG, Ouellette M, Frolkis M, Holt SE, Chiu CP, Morin GB, et al Extension of life-span by introduction of telomerase into normal human cells. Science. 1998;279(5349): 349–352. 9454332 10.1126/science.279.5349.349

[28] Fakhoury J, Marie-Egyptienne DT, Londono-Vallejo JA, Autexier C. Telomeric function of mammalian telomerases at short telomeres. J Cell Sci. 2010;123(Pt 10): 1693–1704. 10.1242/jcs.063636 20427319

[29] Cohen PY, Breuer R, Wallach-Dayan SB. Thy1 up-regulates FasL expression in lung myofibroblasts via Src family kinases. Am J Respir Cell Mol Biol. 2009;40(2): 231–238. 10.1165/rcmb.2007-0348OC 18676775

[30] Jung YJ, Kim JY, Park JH. TGF-beta1 inhibits Fas-mediated apoptosis by regulating surface Fas and cFLIPL expression in human leukaemia/lymphoma cells. International journal of molecular medicine. 2004;13(1): 99–104. 14654978

[31] Hagimoto N, Kuwano K, Nomoto Y, Kunitake R, Hara N. Apoptosis and expression of Fas/Fas ligand mRNA in bleomycin-induced pulmonary fibrosis in mice. Am J Respir Cell Mol Biol. 1997;16(1): 91–101. 8998084 10.1165/ajrcmb.16.1.8998084

[32] Chen C, Akiyama K, Yamaza T, You YO, Xu X, Li B, et al Telomerase governs immunomodulatory properties of mesenchymal stem cells by regulating FAS ligand expression. EMBO molecular medicine. 2014;6(3): 322–334. 10.1002/emmm.201303000 24401839 PMC3958307

[33] Yuan J, Yang BM, Zhong ZH, Shats I, Milyavsky M, Rotter V, et al Upregulation of survivin during immortalization of nontransformed human fibroblasts transduced with telomerase reverse transcriptase. Oncogene. 2009;28(29): 2678–2689. 10.1038/onc.2009.136 19483728

[34] Cha SI, Groshong SD, Frankel SK, Edelman BL, Cosgrove GP, Terry-Powers JL, et al Compartmentalized expression of c-FLIP in lung tissues of patients with idiopathic pulmonary fibrosis. Am J Respir Cell Mol Biol. 2010;42(2): 140–148. 10.1165/rcmb.2008-0419OC 19372246 PMC2822976

[35] Tanaka T, Yoshimi M, Maeyama T, Hagimoto N, Kuwano K, Hara N. Resistance to Fas-mediated apoptosis in human lung fibroblast. Eur Respir J. 2002;20(2): 359–368. 12212968 10.1183/09031936.02.00252602

[36] Armanios MY, Chen JJ, Cogan JD, Alder JK, Ingersoll RG, Markin C, et al Telomerase mutations in families with idiopathic pulmonary fibrosis. N Engl J Med. 2007;356(13): 1317–1326. 17392301 10.1056/NEJMoa066157

[37] Degryse AL, Xu XC, Newman JL, Mitchell DB, Tanjore H, Polosukhin VV, et al Telomerase deficiency does not alter bleomycin-induced fibrosis in mice. Exp Lung Res. 2012;38(3): 124–134. 10.3109/01902148.2012.658148 22394286 PMC4046256

[38] Rudolph KL, Chang S, Millard M, Schreiber-Agus N, DePinho RA. Inhibition of experimental liver cirrhosis in mice by telomerase gene delivery. Science. 2000;287(5456): 1253–1258. 10678830 10.1126/science.287.5456.1253

[39] Lee J, Reddy R, Barsky L, Scholes J, Chen H, Shi W, et al Lung alveolar integrity is compromised by telomere shortening in telomerase-null mice. Am J Physiol Lung Cell Mol Physiol. 2009;296(1): L57–70. 10.1152/ajplung.90411.2008 18952756 PMC2636955

